# Left atrial spherical shape change in atrial fibrillation

**DOI:** 10.1186/1532-429X-16-S1-O41

**Published:** 2014-01-16

**Authors:** Erik T Bieging, Christopher J McGann, Alan Morris, Allen Rassa, Joshua Cates

**Affiliations:** 1 Internal Medicine, University of Utah, Salt Lake City, Utah, USA; 2 Cardiology, University of Utah, Salt Lake City, Utah, USA; 3 Scientific Computing & Imaging Institute, University of Utah, Salt Lake City, Utah, USA; 4 CARMA Center, University of Utah, Salt Lake City, Utah, USA

## Background

Atrial fibrillation (AF) is associated with increase in size of the left atrium (LA). Recent studies have shown that LA shape may be an earlier and independent marker of atrial remodeling in AF. However, previous studies have been limited to qualitative assessments of shape and parameters that do not fully characterize shape. In this study, we use an entropy-driven particle-based shape modeling technique to investigate the relationship between shape and volume of the LA in AF.

## Methods

The Utah AF database was queried for patients who underwent MRI of the LA with paroxysmal AF (n = 50), persistent AF (n = 50), and no arrhythmia (n = 37). The LA walls were segmented manually using Corview (Marrek^®^) software. LA volume was calculated from the segmented slices. The ostia of the pulmonary veins were excluded prior to shape modeling. Based on the method described by Cates et al., a set of 1024 corresponding surface points were mapped onto each LA surface. Principle component analysis (PCA) was conducted to generate 137 parameters (PCA modes). Shape change was observed with respect to the first parameter, and empirically showed increasing spherical shape of the LA. Therefore, this parameter was defined as the "spherical parameter". ANOVA was performed to compare volume and the spherical parameter between control, paroxysmal and persistent groups. Linear regression was used to correlate volume with the spherical parameter in the entire cohort and in each group.

## Results

**Figure 1:**
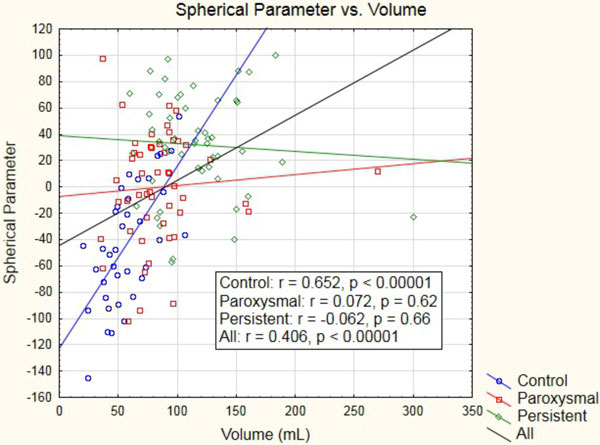
**Volume correlates with the spherical parameter in the entire cohort**. The spherical parameter is strongly correlated with volume in the control group, but is not correlated with volume in the paroxysmal and persistent AF groups.

LA volume was significantly increased in paroxysmals (85.1 mL) relative to controls (57.3 mL), persistents (116.9 mL) to controls, and persistents to paroxysmals (all p < 1 × 10^-3^). The spherical parameter was significantly increased in paroxysmals relative to controls, persistents to controls, and persistents to paroxysmals (all p < 1 × 10^-3^). Linear regression showed significant correlation of volume with the spherical parameter in the entire cohort (p < 1 × 10^-4^) and in the control group (p < 1 × 10^-4^), but no correlation in the paroxysmal and persistent AF groups (Figure 1).

## Conclusions

In this study, we demonstrated that in AF, remodeling of the LA leads to both increased volume and spherical shape change. In non-AF patients, spherical shape change and volume expansion occur together. However in AF patients, spherical shape change occurs independently of volume expansion. Therefore spherical change of the LA, quantified by particle-based shape modeling, may be a useful, unique marker of remodeling in AF.

## Funding

This project was completed with funding from the CARMA center at the University of Utah.

**Figure 2:**
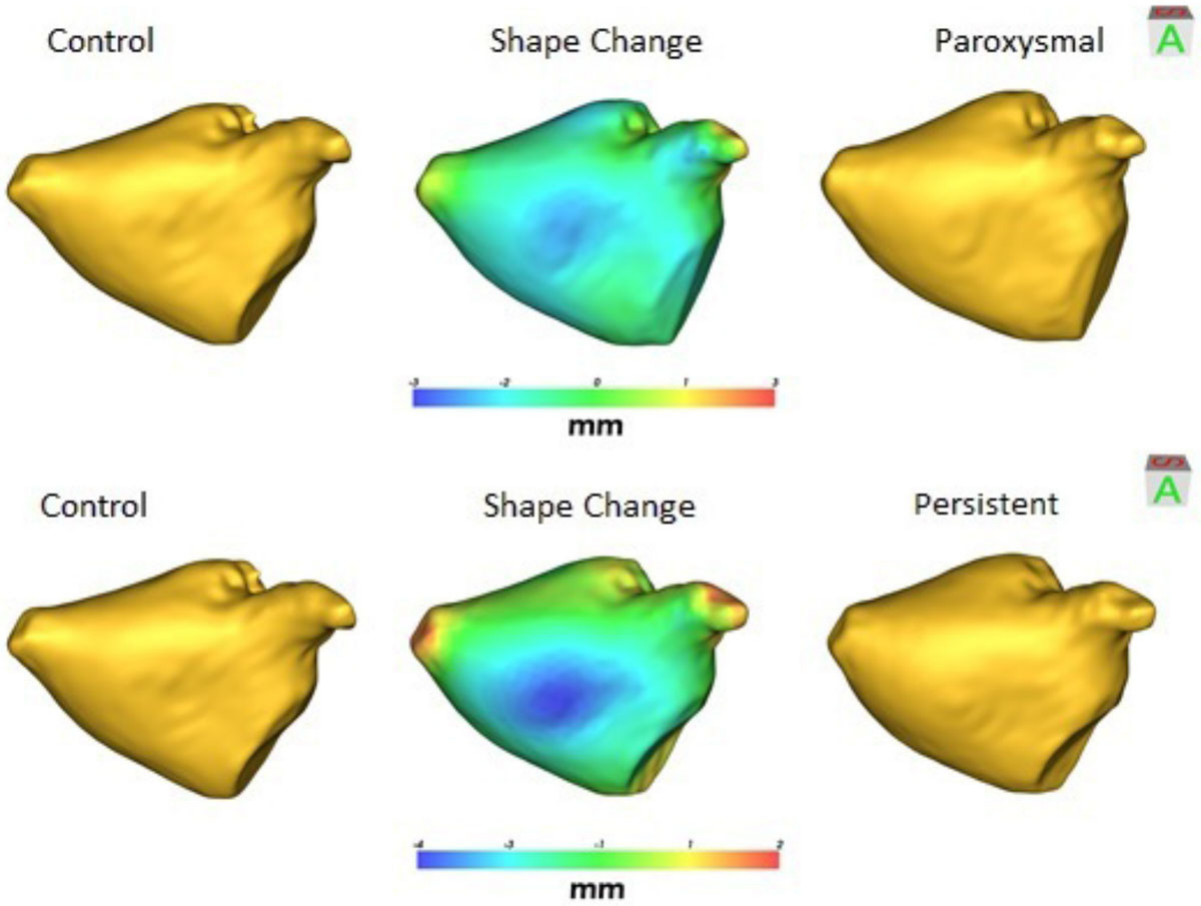
**The mean control (left), paroxysmal (upper right), and persistent (lower right) shapes are shown**. The shape change map shows that the anterior wall balloons outward, and the lateral vein ostia round inward for an overall spherical shape change in paroxysmal and persistent groups, relative to controls.

